# Family history of dementia and brain health in childhood and middle age: a prospective community-based study

**DOI:** 10.1007/s10654-024-01160-2

**Published:** 2024-10-10

**Authors:** Rowina F. Hussainali, Isabel K. Schuurmans, Jendé L. Zijlmans, Charlotte A. M. Cecil, Meike W. Vernooij, Annemarie I. Luik, Ryan L. Muetzel, M. Arfan Ikram, Frank J. Wolters

**Affiliations:** 1 Department of Epidemiology, Erasmus MC University Medical Center Rotterdam, PO Box 2040, Rotterdam, CA 3000 The Netherlands; 2 The Generation R Study Group, Erasmus MC University Medical Center Rotterdam, Rotterdam, The Netherlands; 3 Department of Obstetrics and Gynecology, Erasmus MC University Medical Center Rotterdam, Rotterdam, The Netherlands; 4 Department of Child and Adolescent Psychiatry/Psychology, Erasmus MC University Medical Center Rotterdam, Rotterdam, The Netherlands; 5 Department of Biomedical Data Sciences, Molecular Epidemiology, Leiden University Medical Center, Leiden, The Netherlands; 6 Department of Radiology and Nuclear Medicine & Alzheimer Center Erasmus MC, Erasmus MC University Medical Center Rotterdam, Rotterdam, The Netherlands

**Keywords:** Family history, Dementia, Childhood, Midlife, Brain health

## Abstract

We aimed to determine the association of family history of dementia with structural brain measures and cognitive performance in childhood and mid-life adulthood. We studied 1,259 parents (mean age: 47.3 years, range 31.9–67.4) and 866 of their children (mean age [range] at brain MRI: 9.9 years [8.8–11.9], and for cognition: 13.5 years [12.6–15.8]) of the population-based Generation R Study. Parents filled in a questionnaire on family history, and both parents and children underwent cognitive assessment and neuroimaging. Of all participants, 109 parents (8.6%) reported a parental family history of dementia and 73 children (8.4%) had a grandparental history of dementia with mean age of dementia diagnosis in those affected 75 years (± 7.3). We observed no associations of dementia family history with cognitive ability in either parents or their children, except for worse Purdue pegboard in parents with a parental history of dementia, compared to those without (mean difference [95%CI]: -1.23 [-2.15; -0.31], test range: 21–52). In parents and children, neuroimaging measures did not differ significantly by family history. Results did not depend on age, sex, and *APOE* genotype. Family history of dementia was associated with worse manual dexterity in mid-life adulthood, but not with any other measures of cognitive ability or subclinical brain health in childhood and mid-life. These findings suggest that the association of family history with dementia risk is due chiefly to neurodegenerative rather than neurodevelopmental processes, and might first present with reduced motor skills.

## Introduction

Family history of dementia is an important risk indicator for the development of dementia [1–3]. While rare mutations can give rise to monogenetic dementia within families at a young age, the vast majority of ‘familial’ dementia occurrences relate to polygenic causes of relatively late-onset disease [3]. Timely and targeted prevention is an important area of research to reduce risk in relatives of patients with dementia, but little is known about the specific pathology associated with familial late-onset disease, or the age at which it manifests. As longitudinal studies would take several decades to determine when cognitive trajectories of individuals with a positive family history start to diverge, cross-sectional designs have been applied as a more feasible alternative.

Several studies have turned to cognitive assessments and imaging biomarkers to determine at which age subclinical changes in brain health start to occur with a positive family history. A recent meta-analysis of 34 studies reported that first-degree relatives of patients with late-onset Alzheimer’s disease show cognitive dysfunction (Hedges’ g = -0.16) [4]. Similarly for imaging studies, several studies have found that first-degree relatives of patients with a family history of dementia had an increased risk of small vessel disease, white matter hyperintensities, hypoperfusion, and beta-amyloid and tau [1, 5–7]. As these studies generally focus on older populations in whom some degree of neurodegenerative pathology is likely, they are limited in their ability to distinguish neurodevelopmental from neurodegenerative processes [1, 8, 9]. One paediatric study investigated the effect of dementia family history in 109 children aged 11–16 years, and observed associations with worse memory and global cognition, albeit only in *APOE*-ε4 carriers [10]. Several other paediatric cohorts have studied *APOE* genotype or genetic risk scores for Alzheimer’s disease concerning cognition or brain imaging, but studies have reported inconclusive results for cognition [11, 12], as well as for brain volumes [12–14]. As currently identified genetic risk factors, including *APOE*, explain only 30–40% of familial dementia risk [1], assessment of family history in relation to brain health at younger ages could improve understanding of the timing and nature of hereditary and environmental effects on brain health. Ideally, such studies would use a transgenerational, family study approach to facilitate comparison of hereditary effects across different ages, but such studies are currently unavailable.

We aimed to determine the effect of (grand-)parental family history of dementia on cognitive ability and brain health on structural MRI in children at age 9 (MRI) and at age 13 (cognition) and mid-life adults in a population-based family study. Data were collected within the Generation R study, a prospective community-based birth cohort from Rotterdam, Netherlands, which follows children and their parents from the gestational period into early adulthood.

## Methods

### Study population

The current study is embedded within a prospective population-based birth cohort, the Generation R Study [15]. Women from the city of Rotterdam in the Netherlands, with a delivery date between 2002 and 2006, were invited to participate in the study. Children and both their parents were followed from the gestational period, throughout childhood into early adulthood, with routine visits at certain ages including brain MRI and cognitive assessments [13]. Between 2017 and 2020, a subset of the mothers and their partners were invited for a sub-study that specifically aimed to determine brain health as a means to unravel the ORigins of Alzheimer’s disease aCross the LifE-course: the ORACLE Study [16]. 

Of 3,559 parents invited for the ORACLE Study, 2,084 (58.5%) agreed to participate. They visited the study centre for one day that included questionnaires, blood pressure measurement, cognitive assessment, and brain magnetic resonance imaging (MRI). For the current study, we included all parents who completed the questionnaire on family history. The questionnaire involving family history of dementia was introduced later during the ORACLE study and was available in 1,511 of 2,084 participants (72.5%). We excluded participants who had missing cognitive tests (*n* = 162) and missing sequences on the MRI (*n* = 59). We additionally excluded 31 parents because of motion artefacts or incidental findings on brain MRI (e.g., brain tumour, large cortical infarct), leaving 1,259 parents eligible for the study.

The children of these parents were included if they had undergone brain imaging during their research visit at age 9 (2011–2015), and cognitive testing during their visit at the age of 13 (2016–2020). Of the 1259 parents eligible to participate in this study, 1332 of their children had complete information on brain MRI and cognition available. We excluded 197 children because of low image quality (e.g. motion artefacts) or incidental findings and an additional 269 children were excluded to ensure only one child per family was included in the analysis, leaving 866 (65.0%) children for analysis.

The study was designed in accordance with the guidelines set by the World Medical Association Declaration of Helsinki. The study has been approved by the Medical Ethics Committee of the Erasmus Medical Center, University Medical Center, Rotterdam, the Netherlands. Written informed consent was obtained for all participants. Children aged between 12 and 16, both the child and legal guardian gave consent.

### Data availability

Datasets generated during the current study are not publicly available due to legal and ethical regulations. However, requests for access to the data reported in this paper can be directed to the secretary office of the Generation R Study (secretariat.genr@erasmusmc.nl), in accordance with local, national, and European Union regulations.

### Family history

Family history of dementia was acquired through structured questionnaires administered during an interview [16]. Parents were asked ‘Has your mother or father been diagnosed with dementia?’. The question could be answered with yes, no, or uncertain. If answered yes, parents were asked to provide details about the affected parent, including age at the time of the diagnosis. For the current study, we considered family history positive if a parent reported a diagnosis of dementia in at least one of their parents. For the children, this meant their grandparental family history was positive if either of their parents reported a diagnosis of dementia in at least one grandparent.

### Image acquisition and processing

#### Parent and child structural imaging

For both parent and child, structural magnetic resonance images (MRI) were obtained on a 3T GE Discovery MR750w MRI System (General Electric, Milwaukee, WI, USA) with an 8-channel head coil [16, 17]. The complete procedure has been previously described [16, 17]. In short, we collected T1-weighted images with an inversion recovery-prepared fast spoiled gradient recalled sequence (Tr = 8.77 ms, Te = 3.4 ms, Ti = 600 ms, flip angle = 10 0, Field of view = 220 × 220 mm, acquisition matrix = 220 × 220, slice thickness = 2 mm (1 mm for the children), number of slices = 230). The T1-weighted images were processed through the FreeSurfer analysis suite, version 6.0.0 [18]. Non-brain tissue was removed and the voxel intensities were normalized for B1 inhomogeneity. Next, the images were segmented and all segmentations were manually inspected. The brain measures of interest were the volume of total brain, grey matter, white matter, hippocampal, entorhinal cortex, the middle temporal gyrus, and the parahippocampal gyrus [12, 19, 20]. Volumes across left and right hemispheres were averages, as we did not expect lateralized effects. Intracranial volume (ICV) encompasses the total volume within the skull, including the brain tissue and cerebrospinal fluid spaces.

### Assessment of cerebral small vessel disease

For the parents, volume of white matter hyperintensities (WMH) was acquired from FreeSurfer segmentations. Trained researchers rated all scans for the presence of lacunes and cerebral microbleeds, blinded to familial history of dementia. Lacunes were defined following the STRIVE criteria as focal lesions between ≥ 3 and < 15 mm within the white matter, cerebellum, basal ganglia, or thalamus, as seen on a 2D axial fluid-attenuated inversion recovery (FLAIR) sequence (0.8 × 1.1 × 2.5 mm^3^) and the T1-weighted sequence [21]. Cerebral microbleeds were defined as small hypointense foci with a maximum size of 10 mm on T2*-weighted sequence (0.8 × 1.1 × 1.0 mm^3^).

### Cognition

#### Parents

Parents completed a cognitive test battery as part of the ORACLE study [16]. The battery consisted of six tests, assessing different domains of cognition. A detailed description of the complete test battery can be found elsewhere [16]. Briefly, the assessment included the 15-word learning test [22], the Stroop task [23], the letter-digit substitution test [24], a word fluency test [25], the Purdue pegboard test for manual dexterity [26], and the design organization test [27]. As the latter two tests were introduced later during the study course, they were not offered to 4.5% (design organisation test) and 18.5% (Purdue pegboard) of participants, respectively. We imputed these two cognitive tests (missing completely at random), using a single imputation based on age, sex, education, and other available cognitive tests. To summarize the tests into a single score for global cognition, we computed the g-factor [28] isolating the first component of a principal component analysis, using all six cognitive tests as indicators. The g-factor explained 64.1% of the variance amongst the cognitive tests.

### Children

For children, we assessed the Intelligence Quotient (IQ) as an indicator of cognitive function. During the research centre visit at age 13, children were administered four subtests from the Wechsler Intelligence Scale for Children-Fifth Edition (WISC-V) [29]. Matrix reasoning was used to assess fluid reasoning and was administered digitally. Digit Span (forward, backwards & ranking from high to low) was administered verbally to assess working memory. Symbol substitution was administered digitally, and used to measure processing speed. Finally, Vocabulary was administered verbally, measuring verbal comprehension. All subtest scores were age-normed according to the manual [29]. IQ scores were derived by summing the normed subtests and then converted into IQ scores using a conversion table specifically created for these four subtests by Pearson [30]. 

### APOE genotype

Genotyping in the parents was done by sequencing DNA from blood samples collected during early pregnancy. *APOE* genotype for the parents was determined with a biallelic TaqMan assay (rs7412 and rs429358), and classified in *APOE*-ε4 carriers and non-carriers. Of the total 1259 parents, 1071 (85.0%) had information on *APOE* and of these, 296 (27.6%) had at least one *APOE*-ε4 allele. Genotyping in children was done by sequencing DNA from blood samples obtained from the umbilical cord or with blood samples collected at the age of 6 [31]. *APOE*-ε4 carrier status was determined based on the genotype data. Out of the 866 children, 584 (67.4%) had information on *APOE* and 142 (24.3%) had at least one *APOE*-ε4 allele.

### Other measurements

Information on ethnicity and education was obtained through self-reported questionnaires administered at study inclusion. Because of the relatively small sample size within subgroups, country of origin was categorized for this analysis into Western which includes European, North-American, and Oceanian (as well as Japanese) and non-Western (South America, Central-American, Asia (other than Japan), and African). At the visit of the MRI, height, weight, medication use, and smoking (current smoking, yes or no) were self-reported. BMI was computed from height and weight (kg/m^2^). Systolic and diastolic blood pressure was measured using an automatic sphygmomanometer Omron 907 (OMRON, Matsusaka Co., Ltd., Japan) [32]. Blood pressure was measured two times over a 60-second interval, and individuals with an average systolic blood pressure > 140 mmHg or diastolic blood pressure > 90 mmHg, or the use of blood pressure-lowering medication were classified as hypertensive.

### Statistical analysis

Missing data on non-genetic covariates were imputed through multiple imputation procedures using the package mice [33]. We had 16.7% missing data on education, 13.6% on ethnicity, and 2.1% on hypertension information. As for smoking and BMI, we had less than 0.01% missing data. Data were imputed 20 times (20 iterations) using chained equations and the model estimates for each imputed data set were subsequently pooled using Rubin’s rules [34]. The distribution of covariates was similar in the imputed and non-imputed datasets. All analyses were done in R 3.6.3 [35].

Among the parents, we determined the association of parental history of dementia with brain volumes, using linear regression. Although we expected a low prevalence of small-vessel disease in our population, in an exploratory analysis the association of parental history with the presence of lacunes (yes/no) and microbleeds (yes/no) was determined using logistic regression. All models included age, sex, ethnicity, and ICV (model 1), with further adjustment for educational attainment, hypertension, smoking, BMI (model 2), and *APOE* genotype (model 3). Similarly, for the children, we determined the association of grandparental history of dementia with the same structural brain measures using linear regression. Markers of small-vessel disease were absent in childhood, and therefore not further assessed. All models included age, sex, ICV, and ethnicity (model 1), with further adjustment for *APOE* genotype (model 2).

Next, we determined the association of parental history of dementia with the g-factor and the underlying cognitive tests. Among the children, we determined the association of grandparental history of dementia with IQ and the tests that encompassed IQ, using linear regression models. Models included the same covariates as those for the imaging outcomes.

We performed various sensitivity analyses. First, we stratified the participants by *APOE* ε4 carrier status. Second, we stratified parents at age 50, to account for the fact that some of their parents may still be relatively young for developing dementia. Third, we stratified by age—(grand)parent under or over the age of 80 at diagnosis— based on previously reported increased genetic risk, particularly with younger age at onset [1]. Fourth, we stratified by maternal vs. paternal family history, because previous research has suggested effects may be more profound for maternal than for paternal family history [7, 36]. 

## Results

The population characteristics of the parents and the children are shown in Table 1. In total 1,259 parents and 866 children were included in the study. The mean age of the included parents was 47.3 (± 4.7). Children were on average 9.9 years (± 0.4) during MRI scanning and 13.5 years (± 0.3) at the time of the cognitive assessment. In total, 109 of the parents (8.6%) had a parental history of dementia and 73 children (8.4%) had a grandparental history of dementia. The mean age at dementia diagnosis in affected (grand)parents was 76 years (standard deviation: 7.5 years).

**Table 1:** Characteristics table for the parents and the children

	Parents (*n* = 1,259)	Children (*n* = 866)
Age at brain imaging, years	47.3 ± 4.7	9.9 ± 0.4
Age at cognitive assessment, years	47.3 ± 4.7	13.5 ± 0.3
Sex, female	896 (71.1)	444 (51.2)
European ancestry	1049 (83.4)	608 (70.2)
(Grand)parental family history of dementia	109 (8.6)	73 (8.4)
*APOE*-ε4 carrier	296 (23.5)	142 (24.3)
Educational attainment		
Low	113 (8.9)	n/a
Intermediate	282 (22.3)	n/a
High	653 (51.8)	n/a
Current smoking	148 (11.7)	n/a
Body mass index, kg/m^2^	25.7 (± 4.3)	n/a
Systolic blood pressure, mmHg	126.0 (± 15.4)	n/a
Diastolic blood pressure, mmHg	79.4 (± 10.7)	n/a
Hypertension, n (%)	332 (26.3)	n/a
15-word learning test	9.2 (± 2.7)	n/a
Stroop task	36.0 (± 9.3)	n/a
Letter-digit substitution test	37.2 (± 5.9)	n/a
Word fluency test	27.7 (± 6.4)	n/a
Purdue pegboard test	39.8 (± 4.2)	n/a
Design organization test	38.2 (± 9.0)	n/a
Matrix reasoning	n/a	9.5 (± 2.6)
Digit Span	n/a	9.6 (± 2.7)
Symbol substitution	n/a	13.1 (± 3.2)
Vocabulary	n/a	10.1 (± 2.8)

Values are means (standard deviation) or numbers (percentages); n/a = not applicable. Weight was self-reported

### Cognitive ability in midlife and IQ in childhood

**Fig. 1:**
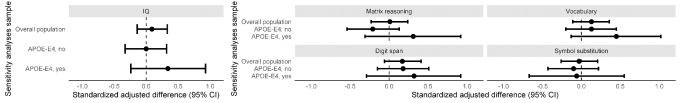
Grandparental history of dementia and cognition in childhood, stratified *APOE* genotype. Legend: This figure shows the standardized adjusted differences (95% Confidence Intervals) in cognitive performance between children with and without a grandparental history of dementia across various cognitive tests and sensitivity analyses

For the parents in mid-life, a parental family history of dementia was not associated with global cognitive performance (g-factor, mean difference [95% CI]: 0.08 [-0.08; 0.26]). Of the various cognitive tests and domains assessed, a positive family history was associated with poorer performance only on the Purdue pegboard task (adjusted mean difference [95% CI]: -1.23 [-2.15; -0.31]; Table 2). These associations were similar for *APOE*-ε4 carriers and non-carriers and did not differ by age of the participant, or age and gender of the affected parent (Fig. 1).

**Table 2:** (Grand)parental history of dementia and cognitive ability in childhood and midlife

	Mean difference (95% CI) Model 1	Mean difference (95% CI) Model 2	Mean difference (95% CI) Model 3
**Cognition in childhood**			
IQ	1.23 (-1.87; 4.34)	n/a	1.23 (-1.87; 4.33)
Matrix reasoning	0.02 (-0.60; 0.64)	n/a	0.02 (-0.60; 0.64)
Digit span	0.47 (-0.17; 1.11)	n/a	0.47 (-0.17; 1.11)
Symbol substitution	-0.09 (-0.86; 0.67)	n/a	-0.09 (-0.86; 0.67)
Vocabulary	0.36 (-0.30; 1.03)	n/a	0.36 (-0.30; 1.03)
**Cognition in mid-life adulthood**			
g-factor	0.11 (-0.06; 0.29)	0.08 (-0.08; 0.26)	0.09 (-0.09; 0.28)
15 word learning test	0.22 (-0.31; 0.76)	0.26 (-0.27; 0.80)	0.08 (-0.49; 0.66)
Stroop test	1.48 (-0.35; 3.31)	1.20 (-0.55; 2.96)	1.54 (-0.31; 3.40)
Letter digit substitution test	-0.85 (-2.00; 0.30)	-0.73 (-1.86; 0.39)	-0.50 (-1.73; 0.71)
Word fluency test	-0.36 (-1.63; 0.89)	-0.31 (-1.58; 0.94)	-0.30 (-1.65; 1.05)
Purdue pegboard test	**-1.34 (-2.26; -0.41)**	**-1.23 (-2.15; -0.31)**	**-1.28 (-2.26; -0.30)**
Design organization test	-0.54 (-2.35; 1.26)	-0.45 (-2.22; 1.31)	-0.92 (-2.83; 0.97)

Model 1: adjusted for age, sex, and ethnicity

Model 2: model 1 with additional adjustment for education attainment, hypertension, BMI, and smoking

Model 3: model 2 with additional adjustment for *APOE* genotype

**Fig. 2:**
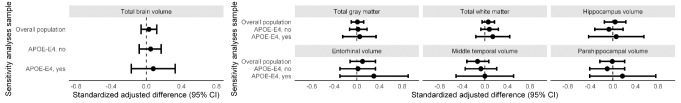
Grandparental history of dementia and brain imaging in childhood, stratified *APOE* genotype. Legend: This figure shows the standardized adjusted differences (95% Confidence Intervals) in brain volumes between children with and without a grandparental history of dementia, across various brain regions and sensitivity analyses

**Fig. 3:**
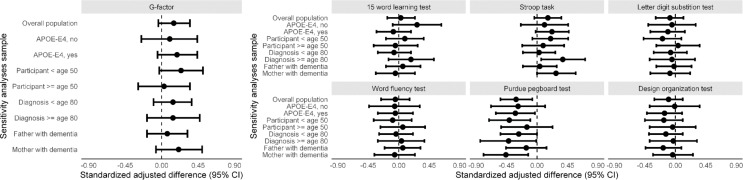
Parental history of dementia and cognitive ability in midlife, stratified by age, *APOE* genotype, and affected parent. Legend: This figure presents the standardized adjusted differences (95% CI) for the g-factor and individual cognitive tests among various sensitivity analysis samples. The vertical dashed line at 0.00 represents no difference, and the black circles indicate the point estimates with horizontal lines representing the 95% confidence intervals

Among the children, grandparental history of dementia was not associated with IQ (mean difference between those with positive history versus those without [95% Confidence Interval]: 1.23 [-1.87; 4.34]), or any of the IQ-score subtests in childhood (Table 2). These results were not modified by *APOE* genotype (Fig. 2).

### Structural brain imaging in midlife and childhood

Parental history of dementia was not associated with any of the volumetric brain measures in midlife (e.g., mean difference in total brain volume [95% CI]: 3.4mL [-5.2;12.1]; Table 3). Of all parents, 41 had at least one lacune (of whom 8 had a positive family history), and 117 had at least one cerebral microbleed (of whom 14 with a positive family history). We did not observe any statistically significant associations between family history and these focal markers of cerebral small-vessel disease, although confidence intervals around the point estimate for lacunes, in particular, were wide (OR [95% CI]: 1.93 [0.76–4.87]; Table 3). Once again, results were not modified by *APOE* genotype and were observed irrespective of age and sex of the affected parent (Fig. 3).

**Table 3:** (Grand)parental history of dementia and brain imaging in childhood and in mid-life adulthood

	Mean difference (95% CI) Model 1	Mean difference (95% CI) Model 2	Mean difference (95% CI) Model 3
**Brain imaging in childhood**			
Total brain volume	2.92 (-6.57; 12,43)	n/a	2.94 (-6.56; 12,45)
Grey matter volume	0.43 (-6.30; 7.18)	n/a	0.45 (-6.29; 7.20)
White matter volume	1.42 (-12.19; 4.07)	n/a	1.42 (-12.20; 4.07)
Hippocampal volume	-0.01 (-0.05; 0.08)	n/a	-0.01 (-0.05; 0.08)
Entorhinal cortex volume	0.04 (-0.04; 0.12)	n/a	0.04 (-0.04; 0.13)
Middle temporal gyrus volume	-0.22 (-0.57; 0.11)	n/a	-0.22 (-0.57; 0.11)
Parahippocampal gyrus volume	-0.00 (-0.06; 0.06)	n/a	-0.00 (-0.06; 0.06)
**Brain imaging in mid-life adulthood**			
Total brain volume	3.75 (-4.87; 12.38)	3.47 (-5.19; 12.13)	5.07 (-4.37; 14.53)
Grey matter volume	1.82 (-3.12; 6.76)	2.15 (-2.84; 7.16)	2.12 (-2.88; 7.14)
White matter volume	0.90 (-2.01; 3.82)	0.85 (-2.08; 3.79)	0.90 (-2.27; 4.09)
Hippocampal volume	-0.00 (-0.06; 0.05)	-0.01 (-0.07; 0.04)	-0.00 (-0.06; 0.06)
Entorhinal cortex volume	-0.00 (-0.06; 0.04)	-0.01 (-0.07; 0.04)	-0.03 (-0.09; 0.03)
Middle temporal gyrus volume	-0.01 (-0.21; 0.17)	-0.01 (-0.21; 0.17)	0.06 (-0.14; 0.27)
Parahippocampal gyrus volume	-0.00 (-0.05; 0.03)	-0.01 (-0.05; 0.03)	-0.00 (-0.05; 0.03)
White matter hypointensity volume (ln-transformed)	0.02 (-0.18; 0.23)	0.05 (-0.15; 0.26)	0.02 (-0.24; 0.21)
Any cerebral microbleeds*	1.05 (0.54; 2.05)	1.09 (0.5; 2.1)	1.09 (0.51; 2.34)
Any lacunes*	2.20 (0.89; 5.47)	1.93 (0.76; 4.87)	n/a

Model 1: adjusted for age, sex, ethnicity, and intracranial volume

Model 2: model 1 with additional adjustment for education attainment, hypertension, BMI, and smoking

Model 3: model 2 with additional adjustment for *APOE* genotype

* All brain volumes are presented as cm^3^, except for white matter hypointensity volume. For presence of microbleeds and lacunes, the effect estimates are odds ratios with corresponding 95% confidence intervals.As only one person with an *APOE*-ε4 allele had a lacune, no statistical analysis could be done

**Fig. 4:**
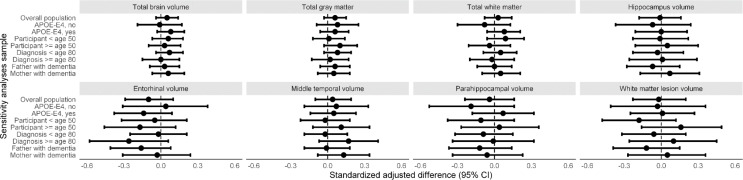
Parental history of dementia and brain imaging in midlife, stratified by age, *APOE* genotype, and affected parent. Legend: This figure presents the standardized adjusted differences (95% CI) for brain volumes among various sensitivity analysis samples. The vertical dashed line at 0.00 represents no difference, and the black circles indicate the point estimates with horizontal lines representing the 95% confidence intervals

Grandparental family history of dementia was not associated with total brain volume or volumes of the cortical grey matter or white matter in childhood (e.g., mean difference in total brain volume [95% CI]: 2.9mL [-6.6;12.4]; Table 3). We observed no association of family history with hippocampal, entorhinal, middle temporal gyrus, and parahippocampal gyrus volumes (Table 3). Results were similar across *APOE* genotypes (Fig. 4).

## Discussion

In this population-based family study of children and their middle-aged parents, we observed few associations between family history of dementia and cognitive ability or structural brain health. Only manual dexterity was reduced in midlife in the parents who had a parental family history of dementia.

Prior studies assessing the association of family history with cognitive ability have mainly been done in somewhat older populations. A recent systematic review and meta-analysis of 34 studies reported that first-degree relatives with a family history of late-onset Alzheimer’s disease perform worse on cognitive assessment than those without a family history [4]. On average, participants were older than in our study (mean 58 years vs. 47 in the current study), which may suggest that a decline in common cognitive tasks related to familial risk may be detected from around the 6th decade of life. Motor function assessment was not part of any of the included studies in the meta-analysis. We have previously shown that worse manual dexterity increases the risk of developing dementia in the general population [37]. As such, the lower dexterity in adults with a positive family history in the current study may reflect the earliest signs of neuropathology, be it of vascular or neurodegenerative nature. Pathophysiological mechanisms may be similar to those underlying previously reported associations of slow gait speed with neuroimaging markers and dementia [38, 39]. Evidently, despite accounting statistically for the multiple comparisons in our study, a type 1 error cannot be ruled out and dexterity findings call for replication.

Previous research indicates an association between family history of dementia in the first degree (parental history of dementia) and brain atrophy [8, 9]. Other studies tried to extend this to imaging markers of cerebral small vessel disease but with inconsistent results [1, 7, 40]. Participants in the abovementioned studies were much older than the current sample, which in midlife can reflect the transition from healthy ageing to the possible preclinical phase of neurodegenerative disease. Our findings indicate that at the average age of 47, we do not observe that a family history of dementia already noticeably impacts subclinical brain health (Table 3). This aligns with a study done by Dounavi et al., who found that APOE carriers and those with a family history of dementia had no prominent macrostructural alterations at the age of 40 to 59 [41]. Nevertheless, it is important to note that the effect estimates for lacunes and to a lesser extent WMH in the current study suggest a potentially meaningful difference between groups, which our study may have been underpowered to detect. More sensitive methods might reveal finer distinctions in brain health at younger ages. These methods could include white matter integrity [42, 43], glucose metabolism [5, 44], and β-amyloid and tau [5, 6].

The observed differences in manual dexterity among middle-aged parents are unlikely to reflect neurodevelopmental processes, as we did not observe any associations between family history and brain health measures in their children. This suggests that while familial factors might influence certain cognitive abilities such as manual dexterity in adults, they do not appear to affect structural brain health in children. The lack of association in the younger cohort reinforces the notion that genetic and environmental influences on brain health may manifest differently across the lifespan. To the best of our knowledge, this is the first study to investigate the grandparental history of dementia concerning volumetric brain measures in childhood. As for cognition, a smaller paediatric study that investigated the effect of family history in 109 children aged 11–16 years, did observe associations with worse memory and global cognition, albeit only in *APOE*-ε4 carriers [10]. Given the substantial contribution of the *APOE* genotype to familial dementia risk, stratification has helped to determine additive genetic interaction and reveal associations of family history with brain health that are otherwise obscured by *APOE* genotype. While certain studies reported stronger associations in *APOE* carriers in mostly older populations [4, 45, 46], other studies did not [4]. In the current study, we observed no effect modification by *APOE* genotype in either children of parents. All in all, this supports the earlier notion that the genetic effects of known Alzheimer genes seem limited in early life [11, 12]. Longitudinal models of brain development in future studies may be more sensitive to detecting diverging brain development concerning familial dementia predisposition.

Strengths of this study include its transgenerational approach to studying family history of dementia and brain health, with extensive data available to correct for potential confounding. There are certain limitations, too. First, we did not have access to medical records of affected (grand)parents to verify the questionnaire information on family history, which may have led to misclassification and dilution of effect estimates. Moreover, some of the grandparents may have developed dementia after the date that we interviewed their children, potentially underestimating the familial burden in some participating families. Second, we were unable to determine the nosological diagnosis of the grandparents and were thus unable to assess for example Alzheimer’s disease-related risk specifically. Third, given the relatively low prevalence of cerebral infarcts in early midlife, our study was underpowered to detect a meaningful association of family history with the prevalence of lacunes and cortical infarcts. Also, the sample size was limited for stratification on *APOE*, and larger studies are needed to provide more robust evidence regarding the interaction between family history of dementia and *APOE* genotype. Fourth, despite adjustment for several lifestyle and social determinants of health confounders, the difference in dexterity may to some extent reflect shared lifestyle rather than genetic predisposition, for example, due to residual confounding with incorrect self-reported weight. Finally, our community-based sample was reflective of an urban population in a Western European country, and findings may not be generalisable to other settings or ancestries.

In conclusion, based on the data analysed here, a family history of all-cause dementia is not associated with cognitive ability or subclinical brain health in childhood and early mid-life, apart from worse manual dexterity in mid-life adulthood. These findings suggest that the association of family history with dementia risk may chiefly be due to neurodegenerative and cerebrovascular rather than neurodevelopmental processes, and might first present with reduced motor skills.

## References

[1] Wolters FJ, van der Lee SJ, Koudstaal PJ, van Duijn CM, Hofman A, Ikram MK, et al. Parental family history of dementia in relation to subclinical brain disease and dementia risk. Neurology. 2017;88(17):1642–9. 28356461 10.1212/WNL.0000000000003871

[2] van der Lee SJ, Wolters FJ, Ikram MK, Hofman A, Ikram MA, Amin N, et al. The effect of APOE and other common genetic variants on the onset of Alzheimer’s disease and dementia: a community-based cohort study. Lancet Neurol. 2018;17(5):434–44. 29555425 10.1016/S1474-4422(18)30053-X

[3] Reitz C, Pericak-Vance MA, Foroud T, Mayeux R. A global view of the genetic basis of Alzheimer disease. Nat Rev Neurol. 2023;19(5):261–77. 37024647 10.1038/s41582-023-00789-z PMC10686263

[4] Ramos AA, Galiano-Castillo N, Machado L. Cognitive Functioning of Unaffected First-degree Relatives of Individuals With Late-onset Alzheimer’s Disease: A Systematic Literature Review and Meta-analysis. Neuropsychol Rev. 2022. 10.1007/s11065-022-09555-2 PMC10770217 36057684

[5] Mosconi L, Rinne JO, Tsui WH, Murray J, Li Y, Glodzik L, et al. Amyloid and metabolic positron emission tomography imaging of cognitively normal adults with Alzheimer’s parents. Neurobiol Aging. 2013;34(1):22–34. 22503001 10.1016/j.neurobiolaging.2012.03.002 PMC3402654

[6] Maye JE, Betensky RA, Gidicsin CM, Locascio J, Becker JA, Pepin L, et al. Maternal dementia age at onset in relation to amyloid burden in non-demented elderly offspring. Neurobiol Aging. 2016;40:61–7. 26973104 10.1016/j.neurobiolaging.2015.12.013 PMC4792089

[7] Stamm BC, Lao PJ, Rizvi B, Colon J, Igwe K, Chesebro AG, et al. Parental history of dementia is Associated with increased small Vessel Cerebrovascular Disease. J Gerontol Biol Sci Med Sci. 2020;75(11):2156–61. 10.1093/gerona/glz291 PMC7566406 31838489

[8] Honea RA, Swerdlow RH, Vidoni ED, Burns JM. Progressive regional atrophy in normal adults with a maternal history of Alzheimer disease. Neurology. 2011;76(9):822–9. 21357834 10.1212/WNL.0b013e31820e7b74 PMC3053329

[9] Okonkwo OC, Xu G, Dowling NM, Bendlin BB, Larue A, Hermann BP, et al. Family history of Alzheimer disease predicts hippocampal atrophy in healthy middle-aged adults. Neurology. 2012;78(22):1769–76. 22592366 10.1212/WNL.0b013e3182583047 PMC3359586

[10] Bloss CS, Delis DC, Salmon DP, Bondi MW. Decreased cognition in children with risk factors for Alzheimer’s disease. Biol Psychiatry. 2008;64(10):904–6. 18722591 10.1016/j.biopsych.2008.07.004 PMC2607139

[11] O’Donoghue MC, Murphy SE, Zamboni G, Nobre AC, Mackay CE. APOE genotype and cognition in healthy individuals at risk of Alzheimer’s disease: a review. Cortex. 2018;104:103–23. 29800787 10.1016/j.cortex.2018.03.025

[12] Lamballais S, Muetzel RL, Ikram MA, Tiemeier H, Vernooij MW, White T, et al. Genetic burden for late-life neurodegenerative disease and its Association with early-life lipids, Brain, Behavior, and Cognition. Front Psychiatry. 2020;11:33. 32116848 10.3389/fpsyt.2020.00033 PMC7018686

[13] Shaw P, Lerch JP, Pruessner JC, Taylor KN, Rose AB, Greenstein D, et al. Cortical morphology in children and adolescents with different apolipoprotein E gene polymorphisms: an observational study. Lancet Neurol. 2007;6(6):494–500. 17509484 10.1016/S1474-4422(07)70106-0

[14] Filippini N, MacIntosh BJ, Hough MG, Goodwin GM, Frisoni GB, Smith SM, et al. Distinct patterns of brain activity in young carriers of the APOE-epsilon4 allele. Proc Natl Acad Sci U S A. 2009;106(17):7209–14. 19357304 10.1073/pnas.0811879106 PMC2678478

[15] Kooijman MN, Kruithof CJ, van Duijn CM, Duijts L, van Franco OH. The Generation R Study: design and cohort update 2017. Eur J Epidemiol. 2016;31(12):1243–64. 28070760 10.1007/s10654-016-0224-9 PMC5233749

[16] Lamballais S, Adank MC, Hussainali RF, Schalekamp-Timmermans S, Vernooij MW, Luik AI et al. Design and overview of the Origins of Alzheimer’s Disease Across the Life course (ORACLE) study. Eur J Epidemiol. 2020. 10.1007/s10654-020-00696-3 PMC7847463 33324997

[17] White T, Muetzel RL, El Marroun H, Blanken LME, Jansen P, Bolhuis K, et al. Paediatric population neuroimaging and the Generation R Study: the second wave. Eur J Epidemiol. 2018;33(1):99–125. 29064008 10.1007/s10654-017-0319-y PMC5803295

[18] Fischl B, FreeSurfer. NeuroImage. 2012;62(2):774–81. 22248573 10.1016/j.neuroimage.2012.01.021 PMC3685476

[19] Jack CR Jr., Knopman DS, Jagust WJ, Petersen RC, Weiner MW, Aisen PS, et al. Tracking pathophysiological processes in Alzheimer’s disease: an updated hypothetical model of dynamic biomarkers. Lancet Neurol. 2013;12(2):207–16. 23332364 10.1016/S1474-4422(12)70291-0 PMC3622225

[20] Dickerson BC, Bakkour A, Salat DH, Feczko E, Pacheco J, Greve DN, et al. The cortical signature of Alzheimer’s disease: regionally specific cortical thinning relates to symptom severity in very mild to mild AD dementia and is detectable in asymptomatic amyloid-positive individuals. Cereb Cortex. 2009;19(3):497–510. 18632739 10.1093/cercor/bhn113 PMC2638813

[21] Wardlaw JM, Smith EE, Biessels GJ, Cordonnier C, Fazekas F, Frayne R, et al. Neuroimaging standards for research into small vessel disease and its contribution to ageing and neurodegeneration. Lancet Neurol. 2013;12(8):822–38. 23867200 10.1016/S1474-4422(13)70124-8 PMC3714437

[22] Van der Elst W, van Boxtel MP, van Breukelen GJ, Jolles J. Rey’s verbal learning test: normative data for 1855 healthy participants aged 24–81 years and the influence of age, sex, education, and mode of presentation. J Int Neuropsychol Soc. 2005;11(3):290–302. 15892905 10.1017/S1355617705050344

[23] Scarpina F, Tagini S. The Stroop Color and Word Test. Front Psychol. 2017;8:557. 28446889 10.3389/fpsyg.2017.00557 PMC5388755

[24] van der Elst W, van Boxtel MP, van Breukelen GJ, Jolles J. The Letter Digit Substitution Test: normative data for 1,858 healthy participants aged 24–81 from the Maastricht Aging Study (MAAS): influence of age, education, and sex. J Clin Exp Neuropsychol. 2006;28(6):998–1009. 16822738 10.1080/13803390591004428

[25] Troyer AK. Normative data for clustering and switching on verbal fluency tasks. J Clin Exp Neuropsychol. 2000;22(3):370–8. 10855044 10.1076/1380-3395(200006)22:3;1-V;FT370

[26] Tiffin J, Asher EJ. The Purdue pegboard; norms and studies of reliability and validity. J Appl Psychol. 1948;32(3):234–47. 18867059 10.1037/h0061266

[27] Killgore WD, Glahn DC, Casasanto DJ. Development and validation of the Design Organization Test (DOT): a rapid screening instrument for assessing visuospatial ability. J Clin Exp Neuropsychol. 2005;27(4):449–59. 15962691 10.1080/13803390490520436

[28] Deary IJ. The stability of intelligence from childhood to old age. Curr Dir Psychol Sci. 2014;23(4):239–45.

[29] Wechsler D. WISC-V: Technical and interpretive manual. Incorporated: NCS Pearson; 2014.

[30] Blok E, Schuurmans IK, Tijburg AJ, Hillegers M, Koopman-Verhoeff ME, Muetzel RL, et al. Cognitive performance in children and adolescents with psychopathology traits: a cross-sectional multicohort study in the general population. Dev Psychopathol. 2023;35(2):926–40. 35249585 10.1017/S0954579422000165

[31] Kruithof CJ, Kooijman MN, van Duijn CM, Franco OH, de Jongste JC, Klaver CC, et al. The Generation R Study: Biobank update 2015. Eur J Epidemiol. 2014;29(12):911–27. 25527369 10.1007/s10654-014-9980-6

[32] Khawaja RA, Qureshi R, Mansure AH, Yahya ME. Validation of Datascope Accutorr Plus using British Hypertension Society (BHS) and Association for the Advancement of Medical Instrumentation (AAMI) protocol guidelines. J Saudi Heart Assoc. 2010;22(1):1–5. 24936124 10.1016/j.jsha.2010.03.001 PMC4051388

[33] Buuren Sv, Groothuis-Oudshoorn K. Mice: Multivariate imputation by chained equations in R. J Stat Softw. 2010:1–68.

[34] Rubin DB. Multiple imputation for nonresponse in surveys. Wiley; 2004.

[35] Team RC. R: A language and environment for statistical computing. 2013.

[36] Berti V, Mosconi L, Glodzik L, Li Y, Murray J, De Santi S, et al. Structural brain changes in normal individuals with a maternal history of Alzheimer’s. Neurobiol Aging. 2011;32(12):e232517–26. 10.1016/j.neurobiolaging.2011.01.001 PMC3115436 21316814

[37] Darweesh SK, Wolters FJ, Hofman A, Stricker BH, Koudstaal PJ, Ikram MA. Simple test of Manual Dexterity can help to identify persons at high risk for neurodegenerative diseases in the community. J Gerontol Biol Sci Med Sci. 2017;72(1):75–81. 10.1093/gerona/glw122 27371953

[38] Gomez GT, Gottesman RF, Gabriel KP, Palta P, Gross AL, Soldan A, et al. The association of motoric cognitive risk with incident dementia and neuroimaging characteristics: the atherosclerosis risk in communities Study. Alzheimers Dement. 2022;18(3):434–44. 34786837 10.1002/alz.12412 PMC10064850

[39] Yaqub A, Darweesh SKL, Dommershuijsen LJ, Vernooij MW, Ikram MK, Wolters FJ, et al. Risk factors, neuroimaging correlates and prognosis of the motoric cognitive risk syndrome: a population-based comparison with mild cognitive impairment. Eur J Neurol. 2022;29(6):1587–99. 35147272 10.1111/ene.15281 PMC9306517

[40] Stefaniak JD, Su L, Mak E, Sheikh-Bahaei N, Wells K, Ritchie K, et al. Cerebral small vessel disease in middle age and genetic predisposition to late-onset Alzheimer’s disease. Alzheimers Dement. 2018;14(2):253–8. 29156222 10.1016/j.jalz.2017.08.017

[41] Dounavi ME, Newton C, Jenkins N, Mak E, Low A, Muniz-Terrera G, et al. Macrostructural brain alterations at midlife are connected to cardiovascular and not inherited risk of future dementia: the PREVENT-Dementia study. J Neurol. 2022;269(8):4299–309. 35279756 10.1007/s00415-022-11061-7 PMC9294019

[42] Bendlin BB, Ries ML, Canu E, Sodhi A, Lazar M, Alexander AL, et al. White matter is altered with parental family history of Alzheimer’s disease. Alzheimers Dement. 2010;6(5):394–403. 20713315 10.1016/j.jalz.2009.11.003 PMC2933285

[43] Gold BT, Powell DK, Andersen AH, Smith CD. Alterations in multiple measures of white matter integrity in normal women at high risk for Alzheimer’s disease. NeuroImage. 2010;52(4):1487–94. 20493952 10.1016/j.neuroimage.2010.05.036 PMC2910213

[44] Mosconi L, Mistur R, Switalski R, Brys M, Glodzik L, Rich K, et al. Declining brain glucose metabolism in normal individuals with a maternal history of Alzheimer disease. Neurology. 2009;72(6):513–20. 19005175 10.1212/01.wnl.0000333247.51383.43 PMC2677512

[45] Huang W, Qiu C, von Strauss E, Winblad B, Fratiglioni L. APOE genotype, family history of dementia, and Alzheimer disease risk: a 6-year follow-up study. Arch Neurol. 2004;61(12):1930–4. 15596614 10.1001/archneur.61.12.1930

[46] Ten Kate M, Sanz-Arigita EJ, Tijms BM, Wink AM, Clerigue M, Garcia-Sebastian M, et al. Impact of APOE-varepsilon4 and family history of dementia on gray matter atrophy in cognitively healthy middle-aged adults. Neurobiol Aging. 2016;38:14–20. 26827639 10.1016/j.neurobiolaging.2015.10.018

